# Mobile Support for Older Adults and Their Caregivers: Dyad Usability Study

**DOI:** 10.2196/12276

**Published:** 2019-05-23

**Authors:** Charlene C Quinn, Sheila Staub, Erik Barr, Ann Gruber-Baldini

**Affiliations:** 1 Division of Gerontology Department of Epidemiology and Public Health University of Maryland School of Medicine Baltimore, MD United States; 2 University of Maryland Marlene & Stewart Greenebaum Comprehensive Cancer Center Baltimore, MD United States; 3 University of Maryland School of Nursing Baltimore, MD United States

**Keywords:** older adult, caregiver, mobile health, patient engagement

## Abstract

**Background:**

Evaluation of digital health applications to support older adults’ independence and family caregiving is needed. Digital health is increasingly providing opportunities for older adults and their family caregivers to educate, engage, and share health information across digital platforms. Few apps have documented evidence of usability by older adults and their caregivers.

**Objective:**

The objective of this study was to determine the usability of a mobile app in a community-based older adult population aged ≥65 years. The app was designed to improve engagement of the patient-informal caregiver team.

**Methods:**

This observational usability study was conducted in participants’ homes and independent living facilities in Baltimore, Maryland. Community-dwelling older adults aged ≥65 years and their caregivers enrolled as a dyad (n=24, 12 dyads). The usability evaluation was a mobile and Web-based app that allowed older adult users to record social and health information and share this information with their caregivers. The older adult-caregiver dyad downloaded the app to a smart phone or accessed the Web version, participated in training and onboarding, and used the app for a 1-month period. Participants responded to weekly surveys sent by app push notifications and to the usability and satisfaction surveys at the end of the study. Participant satisfaction and usability were assessed using the Modified Mobile Application Rating Scale (M-MARS) and the System Usability Scale (SUS).

**Results:**

The final sample comprised 16 people (8 dyads). Responses to the M-MARS were comparable between older adults and caregiver respondents in terms of engagement and functionality. Caregivers rated aesthetics slightly higher (mean 3.7) than older adult participants did (mean 3.3). Although most responses to the SUS were around the mean (2.3-3.4), older adults and their caregivers differed with regard to integration of app features (mean 3.7 vs 2.8) and the need to learn more before using the app (mean 2.3 vs 3.1).

**Conclusions:**

Technology ownership and use among older adults and caregivers was high. Usability and engagement of the mobile app was average. Additional training is recommended for older adults and their caregivers, including that on targeted behaviors for digital health record keeping.

## Introduction

The majority of older adults are cared for at home, and most care is provided by informal caregivers including unpaid family members. According to the Institute of Medicine, at least 17.7 million family caregivers provide assistance to persons aged ≥65 years [1,2]. Family caregivers not living close to aging family members require reliance on additional forms of communication. Studies provide evidence that almost half of the older adults report either needing help or receiving help with routine daily (instrumental) activities of daily living: shopping (90%), making medical appointments (61%), speaking to a doctor (55%), ordering medicine (48%), and keeping track of medicine (49%) [3]. Unmet needs can lead to falls, hospitalizations, emergency department visits, mobility issues, and medication errors [3,4].

Evaluation of the potential of mobile technology is needed to support older adults’ independence and the family’s role in caregiving. Older adults and their identified informal caregivers, together as a dyad, have not been included in technology evaluations. A wide range of new technologies marketed as support for older adults (electronic reminders, motion detectors, and wearable sensors) have promise, but only a few are supported by scientific evidence. Experts in the technology and aging field recommend that research on the safety and effectiveness of mobile technology or devices include input from older users and their caregivers [5-9]. Furthermore, there is a myth that older adults do not use technology [10].

Mobile health (mHealth) is a promising tool for delivering interventions designed to promote self-management but is not well understood in older adults, nor are there well-designed studies on its efficacy and effectiveness. Studies investigating Web-based usability evaluations to promote self-management are inconclusive or demonstrate only moderate effects [11-14]. Few studies demonstrating moderate effect are randomized; include usability evaluations maintaining behavior longer than 6 months; include older adults or minorities; or assess quality of life, which may be more important to older adults than disease control [15-17]. Research demonstrates that current self-management mHealth (apps or internet Web portals) support is associated with dropout within 1-3 months and fails to provide ongoing support or communication with providers when decision making is required outside office visits [18,19]. Exclusion of older adults from large clinical trials evaluating mHealth usability evaluations further underserves this population because of the *myth that older adults do not use technology*. A total of 67% of older adults, including minorities, use the internet (75% use it daily), and smart phone ownership is rapidly growing (42% in 2017) [20,21].

As individuals with technology experience continue to age, mobile technologies will become more accepted or automatic forms of communication [22-26]. However, acceptance and use among older adults are less than those among their counterparts owing to the design, cost, and expected usefulness. Even among current older adult users, differences in technology use and skills are observed for new retirees with workplace technology experience, the young-old (65-74 years old) and old-old (≥75 years old) [27]. The purpose of this research study was to conduct Phase I of a usability evaluation of a new mobile app used by older adults and their caregivers for health, by using a private social network (family or nonfamily informal caregivers) [24]. The Phase II study will evaluate the impact of individual app user information managed through an enterprise dashboard in provider practice settings.

## Methods

### Study Population

Participants (n=24, 12 dyads) were recruited at two independent living facilities in Maryland with community outreach programs, which the participants attended. All older adults lived in their own homes in the community and participated in independent living–sponsored activities (ie, classes in Spanish, falls prevention, and Tai Chi). One dyad (older adult and spouse) lived at the independent living facility. Trained facility site champions identified and obtained permission to contact potential eligible participants who were able to self-identify a caregiver to enroll as a dyad. The study sample was a convenience sample for a usability study. We estimated the sample based on potential for recruitment at the study sites and within the budget limits. A caregiver was broadly defined as a family or nonfamily informal caregiver, identified by the older adult as the primary unpaid person who assisted the older adult, if needed. Eligibility criteria for inclusion of the older adults were age≥65 years, living in the community, ownership of a mobile phone or access to the internet, and ability to pass cognitive screening. Participants were excluded if they had history of substance abuse; had a terminal diagnosis; were undergoing active chemotherapy; had significant vision or hearing impairment; were mute or aphasic; or received a physician’s diagnosis of severe dementia, Alzheimer disease, schizophrenia, bipolar disorder, or major psychosis. There were no inclusion or exclusion criteria for caregivers, except that they were identified by the older adult participant and had access to a mobile phone or the internet in order to use the app. Research staff used an Evaluation to Sign Consent and the Modified Mini-Mental State (3MS) to assess the ability of the potential participant to provide informed consent [25]. The 3MS was used in our Evaluation to Sign Consent method, but not for determining or reporting cognitive status. Four dyads were lost to follow-up. Specific reasons for dropouts were not given to the researchers. Respondents who did not return repeated calls from study staff were excluded from the study.

The Institutional Review Board of the University of Maryland, Baltimore, approved the study protocol (HP-00076904), and written informed consent was obtained from all participants.

### Approach

#### Usability Evaluation

Baseline assessments were conducted face-to-face at the community facility or participant’s home. Participants residing out of the area were mailed study materials and provided consent remotely. Members of the dyad participated in group or individual training on the mobile app and Web portal. For convenience of the older adults and caregivers, participants were offered participation in a group training session or individual training. Training materials were the same for the two educational approaches. The pilot demonstration evaluation was a commercially available mobile app or internet-based portal provided by ICmed, which the Maryland Industrial Partnerships program funded [24]. At the time of this study, the app was newly commercially available but had not been specifically evaluated in an older adult population. The app allows users to create a personal profile and family health history tree; input health information into their profile; receive personalized, evidence-based advice based on the user’s unique health profile; and track and collaborate with designated family members or caregivers [26]. The software is designed to connect individual users and caregivers to care providers, including health systems or care managers, but the study evaluated usability at the individual level. The software is designed for users of varying degrees of health status or frailty: Technically savvy and physically capable users can manage their own profile and collaborate freely with caregivers they select; users with limited technical or physical capabilities can participate where they are capable but can rely on the app design to provide the designated caregiver identical information and notification of every alert or message received by the user; and incapacitated users can delegate themselves to a caregiver’s managed account, in which case the caregiver will be the primary coordinator and communicator using the app. For example, a user may include a future provider appointment in the calendar, which all designated caregivers would be able to view.

The participant was guided through the process of creating a new app account and entering basic demographic information. The dyad individuals were linked using the Family Sharing feature, enabling communication within the app. Participant training concluded by sending a test message to the app team, ensuring the dyad was properly connected. Participants were provided an app-onboarding guide and encouraged to add more information, including health information, using the app at home. Participants completing the 1-month usability evaluation and all surveys were given a US $20 gift card to compensate for their time.

#### Study Measures

Demographic data collected from the older adults and caregivers included a self-reported assessment of ownership and use of technology questions developed by the research team because no standardized survey instruments exist.

Participant engagement was measured by weekly surveys sent via an app push notification developed by providers at the independent living facilities. A push notification is an automated message sent by an app to a user when the app is not open. The purpose of such a notification in this study was to notify users when they were asked to respond to a set of questions. These questions were not standardized tools or measures but questions the independent living communities previously used in printed forms for the community outreach program, and were used to assess whether participants would use the app to respond to the same questions. Two types of weekly surveys were sent to participants. One set of push messages asked if the message was received, with options for “yes” or “no” response. The second set of push messages was developed with study sites to assess how engaged participants are in managing their health. Examples of these survey questions included, “I am fully aware of my current health condition,” “I feel more motivated to take care of my health,” and “I learned how to better monitor my health.” Response categories ranged from strongly agree to strongly disagree. The number of responses to questions were tracked for each of three surveys. At the end of the 1 month of use, participants were mailed two surveys—the Modified Mobile Application Rating Scale (M-MARS) and System Usability Scale (SUS) [28,29]. The M-MARS instrument was modified for this study to assess app quality in three dimensions—engagement, functionality, and aesthetics. All items were rated on a 5-point scale from 1, inadequate to 5, excellent. The original MARS was designed as an app quality–rating tool to be used during the process of app development. In this study, commercial app development was complete; however, the three dimensions of app evaluation were relevant to our understanding of usability. As stated by the MARS developers, “the MARS is an easy-to-use, simple, objective, reliable, and widely applicable measure of app quality, developed by an expert multidisciplinary team. Although the generalizability of the MARS is yet to be tested, the scale can be modified to measure the quality of nonhealth related apps” [16]. Examples of the three dimensions of the M-MARS are as follows: (1) Engagement: fun, interesting, customizable, interactive (eg, sends alerts, messages, reminders, and feedback and enables sharing), and well-targeted to audience. (2) Interest: Is the app interesting to use? For example, did the participant use the education tab? (3) Functionality: app functioning, easy to learn, navigation, flow logic, and design of app. The SUS is a validated (*P*=.92) and calibrated instrument that measures a user’s assessments of usability on multiple dimensions, including effectiveness, efficiency, and satisfaction [30]. Responses are measured on a 5-point scale from 1, strongly disagree to 5, strongly agree [31]. SUS questions are listed in Table 1.

**Table 1:** Evaluation of app usability and engagement among participants at 1 month using the System Usability Scale (1=strongly disagree to 5=strongly agree; n=17). Values are presented as scores.

Characteristic		Older adult (n=9), mean (SD)	Caregiver (n=8), mean (SD)	Total, mean (SD)	*P* value (*t* test)
I think that I would like to use the app frequently		3.2 (1.1)	2.7 (1.3)	2.9 (1.2)	.32
I found the app unnecessarily complex		2.6 (1.2)	3.1 (1.4)	2.8 (1.3)	.38
I thought the app was easy to use		3.4 (1.0)	3.4 (1.6)	3.4 (1.3)	.92
I think that I would need the support of a technical person to use the app		3.1 (1.8)	2.4 (1.7)	2.8 (1.7)	.39
I found the various functions in the app were well integrated		3.7 (0.9)	2.8 (1.3)	3.2 (1.1)	.10
I thought there was too much inconsistency in the app		2.1 (1.1)	2.5 (1.5)	2.3 (1.3)	.54
I would imagine that most people would learn to use the app very quickly		3.6 (1.1)	3.0 (1.5)	3.3 (1.3)	.40
I found the app very cumbersome to use		2.4 (1.3)	2.6 (1.5)	2.5 (1.4)	.80
I felt very confident using the app		3.3 (1.4)	2.9 (1.6)	3.1 (1.5)	.53
I needed to learn a lot of things before I could get going with the app		2.3 (1.1)	3.1 (1.6)	2.7 (1.4)	.24
**MARS^a^** **(1=inadequate to 5=excellent)**					
	Engagement component	2.8 (0.8)	2.8 (0.9)	2.8 (0.8)	.94
	Functionality component	2.6 (1.0)	3.0 (1.4)	2.8 (1.2)	.45
	Aesthetics component	3.3 (2.7)	3.7 (0.9)	3.5 (0.8)	.08

^a^MARS: Mobile Application Rating Scale.

### Statistical Analysis

Descriptive statistics (mean and proportions) are presented for the total sample and according to the type of respondent (adult participant and caregiver). Differences between types of respondents were assessed using *t* tests.

## Results

In this usability study, 20 older adult participants provided permission to be contacted. A total of 24 older adult and caregiver subjects were deemed eligible, and 12 dyads were enrolled (n=24). All participants provided their age, and the mean age was 66.3 (SD 15.2) years overall, 77.8 (SD 4.0) years among the older adults, and 54.8 (SD13.3) years among the caregivers. One older adult participant was ineligible due to inappropriate age (<65 years old), and one older adult participant was ineligible due to a diagnosis of dementia. One additional caregiver and one older adult (from different dyads) did not respond to the researchers’ contact attempts and were considered to be lost to follow-up. In total, four dyads were lost to follow-up. Finally, eight dyads (n=16) completed the study.

More women (n=19) than men (n=5) were enrolled (Table 2). Caregivers were predominantly female (n=11, 91.7%). The relationship between the dyads was parent-child in 75% of the participants. There was an equal number of black (n=12) and white (n=12) participants. Over half of the study population reported having at least a college degree or higher education (75%). Income for the majority of older adults enrolled in the study ranged from US $20,000-29,999 (33.3%), whereas the majority of caregivers enrolled had incomes of ≥US $100,000 (data not shown).

Technology skill and use reported by older adults and caregivers at baseline was high (Table 3). All participants used technology for various social and home activities (ie, paying bills) and all, except one, had internet or Wi-Fi at home. Two participants did not own a smart phone and, instead, accessed the internet and app through a Wi-Fi–enabled tablet device. In addition, 75% of older adults reported that they are at least somewhat skillful with technology and electronics, and all 12 caregivers rated themselves at least somewhat skillful; four caregivers considered themselves very skillful (Table 3).

Activities completed by older adults on the phone daily included making calls (75%) and reading emails (58.3%), whereas caregivers reported making calls (91.7%), sending and receiving text messages (91.7%), connecting to the internet (91.7%), and reading emails (91.7%) daily. Older adults in the study preferred to access the internet via a desktop or laptop computer (33.3%) and caregivers accessed the internet on their phone (75%). Common internet activities for both older adult and caregiver participants included connecting with family and friends, keeping up with current events, looking up information, and reading emails. Caregivers reported using the internet for paying bills, making reservations, sending or receiving photos, and purchasing products and services more often per month than older adults.

**Table 2:** Baseline characteristics of the participants and caregivers (n=24).

Characteristic		Older adults, n (%)	Caregivers, n (%)	Total, n (%)
**Sex**				
	Female	8 (67)	11 (92)	19 (79)
	Male	4 (33)	1 (8)	5 (21)
**Race**				
	Black	6 (50)	6 (50)	12 (50)
	White	6 (50)	6 (50)	12 (50)
**Ethnicity**				
	Hispanic or Latino	1 (8)	1 (8)	2 (8)
	Not Hispanic/Latino	11 (92)	11 (92)	22 (92)
**Education**				
	Business/some college/graduate	8 (67)	6 (50)	14 (58)
	Graduate school	4 (33)	6 (50)	10 (42)
**Marital status**				
	Married	6 (50)	8 (67)	14 (58)
	Widowed	3 (25)	0 (0)	3 (13)
	Divorced	2 (17)	3 (25)	5 (21)
	Never married	0 (0)	1 (8)	1 (4)
	Missing	1 (8)	0 (0)	1 (4)
**Relationship to the other (caregiver or older adult participant)**				
	Spouse	2 (17)	2 (17)	4 (17)
	Child	0 (0)	9 (75)	9 (38)
	Parent	9 (75)	0 (0)	9 (38)
	Friend	1 (8)	1 (8)	2 (8)
**Distance to relative who can provide assistance**				
	<25 miles	6 (50)	6 (50)	12 (50)
	25-50 miles	2 (17)	2 (17)	4 (17)
	>50 miles	2 (17)	2 (17)	4 (17)
	Would need an airplane	2 (17)	2 (17)	4 (17)
**Currently living with the other (caregiver or older adult participant)**				
	No	10 (83)	10 (83)	20 (83)
	Yes	2 (17)	2 (17)	4 (17)

**Table 3:** Baseline technology ownership and use (n=24).

Component			Older adults (N=12), n (%)	Caregivers (N=12), n (%)
**Level of skillfulness with technology and electronics**				
	Not skillful at all/not very skillful		3 (25)	0 (0)
	Somewhat skillful		6 (50)	2 (17)
	Pretty skillful		3 (25)	6 (50)
**Internet or Wi-Fi at home**				
	No		1 (8)	1 (8)
	Yes		11 (92)	11 (92)
**Use of internet for the following activities**				
	**Connecting with family or friends**			
		Never	1 (8)	1 (8)
		<Once per month	1 (8)	1 (8)
		1-5 times per week	1 (8)	3 (25)
		Every day or almost every day	8 (67)	7 (58)
	**Keeping up with current events**			
		Never	1 (8)	1 (8)
		<Once per month	1 (8)	0 (0)
		1-5 times per week	0 (0)	1 (8)
		Every day or almost every day	9 (75)	10 (83)
	**Looking for information**			
		Never	1 (8)	0 (0)
		<Once per month	0 (0)	0 (0)
		1-5 times per week	2 (17)	2 (7)
		Every day or almost every day	8 (67)	10 (83)
	**Paying bills**			
		Never	4 (33)	0 (0)
		<Once per month	6 (50)	5 (42)
		1-5 times per week	1 (8)	5 (42)
		Every day or almost every day	0 (0)	2 (17)
	**Reading emails**			
		Never	2 (17)	0 (0)
		<Once per month	1 (8)	0 (0)
		1-5 times per week	0 (0)	1 (8)
		Every day or almost every day	7 (58)	11 (92)
**Devices used most to access internet**				
	Desktop computer		2 (17)	2 (17)
	Laptop computer		2 (17)	1 (8)
	Computer tablet		3 (25)	0 (0)
	Phone		5 (42)	9 (75)

The push survey messages received an average of 52%-57% responses from the participants. Response rates from older adult participants decreased from 56% to 46%, whereas those from caregivers remained at 58% over the 1-month period (data not shown in table). In the first push survey (week 1), participants strongly agreed that they were fully aware of their health conditions (66.7%), wanted to learn how to take care of their health (50%), and felt motivated to take care of their health (66.7%). In week 4 of the intervention, among participants responding, “strongly agree,” 60% were aware of their health conditions, 40% wanted to learn how to take care of their health, and 40% felt motivated to take care of their health. Fifty percent of caregivers indicated they wanted to use the app to “manage my or my loved one’s health appointments or records” and “use the information to discuss or share health information” and 67% wanted to become “more engaged in their love one’s health and have access to information in one place.”

The SUS was administered to assess the usability of the mobile app, and specific SUS questions are included in Table 1. Although most responses fell along the scale midpoint (response of 3), older adults and caregivers differed with regard to several responses. Older adults more likely considered the app functions to be well integrated compared to caregivers (mean 3.7 vs 2.8; *P*=.10). Fewer older adults felt they needed to learn a lot of things before they could use the mobile app as compared to the caregivers who responded (mean 2.3 vs 3.1; *P*=.24). Responses to the M-MARS were comparable between older adult and caregiver respondents on engagement and functionality measures. Caregivers rated the aesthetic component slightly higher (mean 3.7) than older adult participants did (mean 3.3; *P*=.08).

## Discussion

### Principal Findings

Smartphone ownership and use among older adults has increased with nearly four in ten owners, doubling in number since 2013 [21]. This usability study revealed that while technology use was common in the cohort among well-educated older adults, engagement with the mobile app was average. Studies have reported older adults and caregivers may benefit from additional technical device training given either as a group or one-on-one [32-35]. In this study, participants were guided through onboarding training and provided an onboarding guide for self-use at home; participants would have probably demonstrated prolonged engagement if they were given weekly training sessions or reminders for engagement. Smartphone ownership was high among study participants, and the low rating for “needing to learn a lot of things before could get going with the app” indicates that participants could use the app (Table 1).

Older adult study participants were asked to change their behavior about how they store and manage health records by entering basic health information into the app. Previous research has demonstrated that older adults experience significant difficulties in using personal health record systems to complete simple health management tasks and are significantly less likely to use patient portals [36-38]. The research reported here identifies several areas where technology may be beneficial for older adult users. Communication technology (electronic health or mHealth) like the app evaluated in this study may be used for older adults to improve participation in health care decisions made by informal caregivers and providers [39], to self-manage health and social needs [40], and to improve engagement and social connections [41,42]. The study results are consistent with those of our previous research documenting that older adults will use mHealth to monitor or self-manage a specific disease like diabetes, because there is a perceived need for monitoring [43,44]. App use for this study may have been affected by the perceived lack of need for such an app. Caregiving experts stress the importance of creating a centralized health-related communication hub, given the complexity of medical conditions and volume of documentation that accumulates in the care of older adults [45]. In our study, we approached older adults first and asked them to enlist their caregiver to participate in the study. One possible approach to engaging older adults is to first establish the commitment of their caregiver. Studies demonstrate that caregivers recognize the need for digital information sharing and want to be informed of their loved one’s medical care [7,8]. Caregivers are more likely to recognize the need for health record management and may be motivated to recruit and engage older adults to use mobile apps while also serving as the app account manager. App developers may alternatively consider targeting the “enterprise”—service providers such as long-term care facilities, assisted living facilities, or physicians’ offices—rather than community members directly. The enterprise connection may be more appropriate because the app could be designed to link into electronic records, alleviating user burden. Although in our study, the independent living community champions assisted in study recruitment, the independent living facilities were not directly communicating with potential older adults as an enterprise approach, including assistance with onboarding and maintaining community members’ records.

The pilot study design provided researchers and app developers with valuable information to improve usability of the app. Participants needed a dedicated and responsive support line for technical issues or user-attributed problems with the app. Technical issues, including, but not limited to, log in and connectivity issues, discouraged participants and delayed or limited use, likely leading to loss to follow-up. Links to education sites embedded within the app need to be specific to the health issues of older adults. Additionally, users may remain better engaged with customization, such as greeting users by showing their name on the welcome screen and as they navigate through the app’s features.

The results of this evaluation are limited to the target study population. Participants were willing to enroll in a research project and participate in the study had to own a mobile phone, tablet, or device to access the internet. The cohort may not be representative of the current population of older adults who do not use digital resources. As younger generations age, technology experience and use will be ubiquitous. Additionally, older adult participants in the study had to identify a caregiver to participate in the study, which posed a recurrent issue: Not all older adults have an immediate caregiver or are willing to define a person’s role as “caregiver.” Researchers attempted to address this concern by using alternate phrases such as “loved one” or “care partner.”

### Conclusions

This usability study of a mobile and Web-based app in community-dwelling older adults and their caregivers demonstrated that technology use is high among this population; however, data indicated low participant usability and engagement. Mobile app companies would benefit from including older adults and caregivers in the development of technologies aimed at behavior change, including changes in behavior to maintain health records. This study provides information on the usability of a mobile app to support older people and their caregivers. The study further demonstrates the importance of education and training on technology use for older adults and their caregivers. Caregivers with technology experience may play an important role in demonstrating the use and benefits of technology to support care of older adults.

## Abbreviations

mHealth: mobile health
M-MARS: Modified Mobile Application Rating Scale
SUS: System Usability Scale
3MS: Modified Mini-Mental State
